# Promoting Social and Emotional Learning and Subjective Well-Being: Impact of the “Aislados” Intervention Program in Adolescents

**DOI:** 10.3390/ijerph17020609

**Published:** 2020-01-17

**Authors:** Javier Cejudo, Lidia Losada, Roberto Feltrero

**Affiliations:** 1 Faculty of Education of Ciudad Real, University of Castilla-La Mancha, 13071 Ciudad Real, Spain; manueljavier.cejudo@uclm.es; 2 Department of Research Methods and Assessment in Education II, Faculty of Education, UNED, Juan del Rosal, 14, 28040 Madrid, Spain; 3 Higher Teacher Training Institute “Salomé Ureña”, Calle Caonabo, Esquina Leonardo D’Vinci, Santo Domingo 10114, Dominican Republic; rfeltrero@fsof.uned.es

**Keywords:** social and emotional learning, video game, gamification, adolescence, secondary education, well-being

## Abstract

The aim of this study is to experimentally assess the effects of an intervention program through a video game called “Aislados” for the improvement of subjective well-being, mental health and trait emotional intelligence of a sample of adolescents (*n* = 187). We used well-established measures with appropriate psychometric properties. The study used a quasi-experimental design of pre-test/post-test repeated measurements with a control group. First, a multivariate analysis of variance (MANOVA) and then descriptive analyses and variance analyses (ANOVAs) were carried out by the adolescents randomly assigned to the experimental and control conditions. Then, a multivariate analysis of covariance (MANCOVA) was performed on the study’s variables as a whole. Descriptive and covariance analyses of the post-test scores were carried out (ANCOVAs post-test, co-varying pre-test scores), in order to demonstrate the impact of the program. The effect size was reckoned (Cohen’s *d*). The results confirm statistically-significant differences in: Health-Related Quality of life, positive affect and mental health. The study provides an effective intervention tool which has been experimentally validated. The overall results allow for emphasizing the importance of the implementation of programs aimed at encouraging social and emotional learning throughout adolescence as protective resources in fostering emotional and behavioral adjustment in adolescents.

## 1. Introduction

The aim of education is the integral development of the personality of the students, promoting cognitive, personal and social competencies. Therefore, schools should not only promote the academic growth of students but also their personal growth [1,2]. Some associations with a global impact (e.g., Collaborative for Academic, Social, and Emotional Learning – CASEL, World Health Organization – WHO) emphasize the importance of social competence and social-emotional well-being in the development of children and adolescents [3].

The European Council considers personal and social skills and learning to learn as key competencies for a successful life in society and of equal importance to the other seven key competencies such as digital or multilingual competencies [4]. Research has shown that the promotion of social and emotional skills in educational contexts provides benefits to adolescent students (e.g., [5]), including a critical aspect during the whole period of schooling, such as academic performance [6,7]. Domitrovich et al. [8] have defined social and emotional learning (SEL) as a process through which social-emotional competence develops in five domains: self-awareness, self-management, social awareness, relationships skills, and responsible decision making [1]. The programs which purpose is to develop SEL focus on these five competencies [9]. Weissberg et al. [9] show how SEL also promotes intrapersonal, interpersonal and cognitive competencies for cognitive development.

In addition, the WHO defines SEL as a collection of life skills [10] and, therefore, is conceived as a potential protective factor and promoter of mental health [11]. In the same way, SEL programs are one of the most successful interventions to promote the integral and positive development of students [1]. The fundamental purpose of SEL is the promotion of the quality of life and well-being of people [8,12]. Promoting the social and emotional well-being of young people is a determining factor in their positive development, which allows them to achieve positive results in school, work and in life in general [1].

The evaluation of the effectiveness of the SEL programs shows positive results on academic achievement of the students, on mental health, on psychological and social adjustment and on positive behaviors in health [1,3,8,9,13,14]. Taylor et al. [15] conclude that participants obtained better results than the control group on socio-emotional skills and indicators of psychological well-being. Thesefindings show that programs based on SEL have a similar impact on children and adolescents [8]. For the aim of this study, it must be pointed out that mental health problems in secondary school are considered as serious public health issues. At that age, mental problems can limit student’s abilities to develop positive relationships to their environment and, in this way, can also limit their academic and social performance. In this regard, some studies prove that SEL key competencies can prevent such student difficulties [16,17].

Among the different strategies that can be used to introduce and teach social emotional learning skills to kids and young people, games are a good option to create fun environments to engage them in learning activities that help kids become more self-aware, develop positive relationships, show empathy towards others, manage emotions, use self-control, resolve conflicts, and make positive decision [18]. Insofar as most today games for kids are electronic games, it is also a good strategy to deploy that game strategies using software technologies. 

Video games are one of the most popular recreational activities among young people nowadays [19]. Exploring the boundaries between use and abuse of video games, it can be found studies that point out the negative and positive psychological impact of video games. The negative impact of video games on the psychological well-being and social life of gamers [20] has been proven by studies that associate poor quality interpersonal relationships, dissatisfaction with life and loneliness with the use and abuse of video games [21,22]. Other studies show correlations between video game abuse and emotional and behavioral problems in young people [23] including poor sleep [24], depression and anxiety [25]. 

In the other hand, this negative impact of video games has been questioned in recent studies [26,27,28] that are focused on the potential of video games to improve cognitive and social learning. In this regard, some data indicates that the use of video games in gamification strategies can positively affect learning and teaching processes [29,30,31,32] and cognitive, motivational, emotional and social processes [33]. Those results show how educational technologies can offer a new path for the development of emotional skills that are, indeed, a new challenge for traditional learning strategies. In this sense, video games and computer simulations based on physical activity are nowadays widely used as innovative strategies for training several sports. For instance, there are some evidences that prove how video games based on physical activity have positive effects on self-appreciation or well-being [34], as well as on general mood [35]. Those computational games are becoming a new sport discipline by itself, becoming a field of study into sports psychology, and generating new ideas for the treatment of psychological problems. For instance, computer videogames, smartphone applications and more developed similar tools, as immersive virtual reality environments, are being studied nowadays as therapeutic resources for emotional problems related to anxiety [36]. The development of such computational environments of augmented reality could also promote improvements on important academic areas for adolescents as writing [37], math [38] or language learning [39].

Focusing on emotional intelligence (EI) and well-being, the use of video games among adolescents, like popular Pokemon Go, has been confirmed as a tool for the enhancement in selective attention, concentration and sociability [21] and, in this way, improving cognitive performance and EI. Other studies conclude that good strategies involving video games and computer simulations can improve life satisfaction and prosocial behavior reducing the probability of suffering emotional problem [40,41]. Consequently, it has been previously justified the aim of this study, that is to say, the analysis of the possible benefits of the use of video games by children and adolescents in more detail, specifically in the context of educational strategies for improving EI, subjective well-being, health and life satisfaction.

There are examples of successfully application of video games educational strategies for the improvement of socioemotional skills. For instance, the application of the video game “Spock” show how the emotional intelligence capacity [42] and the psychosocial adjustment [43] have been improved on experimental groups of adolescents. In addition, D’Amico [44] has shown the efficacy of the training performed with that kind of software improving performance in emotional tasks in a group of children with ages ranging from 8–12.

The current study presents an intervention program called “Aislados” that considers the theoretical assumptions of Social and Emotional Learning (SEL) by using video games as an educational tool. “Aislados” is an educational program for middle and high school students developed in 2016 by the Interdisciplinary Service of Attention to Drug Dependencies (in Spanish, SIAD) and founded by National Spanish Plan against Drug addictions (http://www.aislados.es/ financed by the Government Delegation for the National Plan on Drugs). It has been designed as a protective resource for adolescents by means of improving their psychological well-being and empowering them to avoid young risk behaviors related to addictions, violence or emotional disorders. This strategy has been recently proven by the evidences that show how emotional indicators like self-esteem, quality of interpersonal relationships, control of life events, and management of negative emotions, can lead to depression and social and educational impairment, becoming an important risk factor for substance misuse [45,46]. 

Following innovative gamification strategies, as video games and role-playing games, they designed a framework for teaching social, cognitive and emotional skills (life-skills). Both games, video game and role-playing game, are based in the same principles and educational structure and has to be completed by educational sessions driven by teachers. Video game and role-game are educational strategies very suitable for SEL interventions as stated by Weissberg et al. [9] when they point out how important is to apply and improve motivational strategies for those interventions. 

“Aislados” video game consists of a set of virtual characters deployed in an imaginary boat traveling to some newly discovered islands (a descriptive video can be seen at https://www.youtube.com/watch?v=I6N41WREYbY&list=PLLrCXpyb6EFLnQWoDScEc33VsUqPE-5kx&index=3 (Spanish with English subtitles available through YouTube translation). The player interacts with those characters getting involved in conversations to get information for task achievement or solving riddles that are included in the video game. Thus, the “Aislados” video game provides a virtual environment where the main goal of students is to reach a full understanding of the character’s personalities and emotional states, developing positive affect towards characters and deploying emotional intelligence to understand the problems and situations involved at every conversation or interaction. Very interestingly, those activities are being held individually by students, in a way that makes more interactions possible and a more flexible environment for each student to develop their own emotional strategies to solve game tasks, which is an opportunity for the personalization of the learning process of this type of socio-emotional competencies. The intervention program is complemented through explicit instruction (sessions) by teachers for full understanding of emotional situations and decisions taken by players (students) during the game. Thus, the idea that SEL-based programs should encourage activities both inside and outside the classroom, including reaching the community, is emphasized [1]. 

“Aislados” is focused on improving social competence and social-emotional well-being in adolescents and young people, the nuclear objectives of the associations that promote SEL. In this regard, an indicator of social-emotional well-being is subjective well-being (SWB): the cognitive component (satisfaction with life) and the affective component (positive and negative affect) [47,48]. The cognitive component of SWB (CWB) is based on beliefs and judgments about one’s life [49]. While the affective component of well-being (AWB) implies an individual hedonistic balance, that is, how often do people experience positive and negative emotions [48].

Currently psychological research is interested in the study of how positive psychological variables affect personal development [50]; this is the theoretical framework named as positive psychology [51]. Among the positive psychological variables, one of the most scientifically supported is emotional intelligence [52,53]. Despite the controversies surrounding the concept of emotional intelligence [54] it can be defined as the set of individual differences in the identification, expression, use, comprehension and regulation of one’s own emotions and emotions [55]. Emotional intelligence has also close relationships with the SWB and physical and mental health variables [56,57]. Specifically, emotional intelligence as a trait has the highest positive correlations with SWB [58]. These cited studies prove that an efficient management of the components of emotional intelligence can promote positive emotional states and a reduction of negative emotional states, promoting a greater SWB [57].

In sum, the aim of research is to assess the effects of this intervention program for the improvement of SWB, mental health and trait emotional intelligence among a sample of Spanish adolescents of middle and high school age. We adopted a quantitative approach to explore the following specific hypotheses regarding the impact of “Aislados” Program on Spanish adolescents: (1) It will enhance the SWB; (2) It will increase mental health (MH); (3) It will increase trait EI (TEI).

## 2. Materials and Methods 

### 2.1. Participants

The sample has been made up of 187 adolescents, aged 12 to 17 (M = 13.82 SD = 1.62). A non-probabilistic sampling was used, but subjects are assigned at random to the experimental (*n* = 97) and control (*n* = 90) condition in the pre-test stage. The data were provided by middle and high school students (53% girls). 

All students took part voluntarily in this study. The intervention was a scheduled activity during their timetable. This study was carried out in accordance with the Declaration of Helsinki and ethical guidelines and was approved by the Research Ethics Committee of the UNED. Participants’ parents gave informed written consent and adolescents gave verbal assent. Moreover, special permissions where given by the school management team.

Parental consent was used as the main inclusion criterion. Some students were excluded according to the following exclusion criteria: (a) students previously removed from school for disciplinary reasons; (b) students with special educational needs related to intellectual disability; and (c) students not attending at least 75% of the intervention programme sessions. 

### 2.2. Measures

In this study, we used well-established measures with appropriate psychometric properties (see Table 1).

#### 2.2.1. Health-Related Quality of Life Questionnaire for Children and Young People (KIDSCREEN); Kidscreen-10 Index [59]. 

This self-report version contains 10 items to assess subjective Health-Related Quality of Life (HRQL) and well-being; it provides an index of global HRQoL covering physical, psychological and social facets. It is related to the cognitive component of well-being. The questionnaire can be completed by children and adolescents aged from 8 to 18 years. Questions are on 5-point Likert scales from “never” to “always” or from “not at all” to “extremely”. 

#### 2.2.2. Satisfaction with Life Scale (SWLS) [60,61]. 

SWLS is a short 5-item self-report questionnaire where people judge whether their life is satisfying on a 7-point rating scale, from 1 = strongly disagree to 7 = strongly agree. The scale provides a global measure of life satisfaction. It is related to the cognitive component of well-being.

#### 2.2.3. Positive and Negative Affect Schedule (PANAS) [62,63]. 

PANAS is a self- reported adjective checklist designed for the assessment of 20 different feelings and emotions. It contains two subscales with ten items each, representing two constructs: positive affect (i.e., active, attention, determined, excited, inspired, interested…) and negative affect (i.e., afraid, guilty, hostile, irritable, upset). It is related to the affective component of well-being. Participants used a 5-point scale ranging from 1 = ‘‘very slightly or not at all’’ to 5 = ‘‘extremely’’.

#### 2.2.4. Mental Health (MH-5) [64,65]. 

MH-5 is one of the subscales of the SF-36 Health Questionnaire. MH-5 is a short 5-item self-report questionnaire that assesses general mental health, specifically depressive and anxious symptomatology in the past month. It is related to the cognitive component of well-being. Participants respond to a 6-point rating scale, ranging from 1 (always) to 6 (never). 

#### 2.2.5. Trait Emotional Intelligence Questionnaire Adolescents Short Form (TEIQue-ASF) [53,66].

It is a self-report inventory designed to measure global trait emotional intelligence which contains 30 items, with Likert scale response options ranging from (1) “Completely disagree” to (7) “Completely agree”. The measure provides a total score which is obtained by adding up the 30 items. 

### 2.3. Procedure

The study followed a quasi-experimental design of repeated measures (pre-test and post-test) including a control group, where following variables were assessed: Health-Related Quality of Life (KIDSCREEN), Satisfaction with Life (SWLS), Positive Affect (PA) and Negative Affect (NA), Mental Health (MH), Trait Emotional Intelligence (TEI). 

Students were requested to complete anonymously the measures mentioned before. All participants were assured that the data would be kept confidential and would be used for research purposes only. Specifically, the participants of the experimental group were informed of the purpose of the investigation.

At first, the tests were applied to the experimental and control groups. Subsequently, the intervention program was implemented in experimental groups during the school day and within school schedule. In the following academic year, after the evaluation of the effects of the program, the control group participated in the program in the same way as experimental group; within the Tutorial Action Plan of the Guidance Department, whereby activities usually focus on social skills and guidance, personal, academic and professional. Specifically, the intervention consisted of 28 h-long sessions, with duration of 55 min each, carried out weekly during a school year. The sessions were carried out during the tutoring hour in secondary education (weekly timetable). The program was implemented by secondary teachers who voluntarily decided to participate in “Aislados” training. After the intervention, in the post-test stage, pretest measures were administered again to the experimental and control groups. Control groups received the same intervention after the study.

Teachers received official training supported by the Department of Education through a 10-h workshop about the implementation of the program. In addition, weekly coordination meetings were held to agree on the development of each of the sessions in class, as well as the explanation of the instructions of the video game to the students (during the tutorial coordination time).

#### Training Program Description

The video game “Aislados” is inspired by the four elements that facilitate the implementation of programs according to The Collaborative for Academic, Social, and Emotional Learning (CASEL): (1) Sequenced (connected and coordinated activities to foster skills development); (2) Active (active forms of learning to help students master new skills); (3) Focused (containing a component that emphasizes developing social and emotional skills); (4) Explicit (targeting specific social and emotional skills) [7].

The “Aislados” video game includes interactive activities that aim to improve socio-emotional skills. The video game presents hypothetical situations that require the implementation of intrapersonal and interpersonal competencies. Students have to decide which answer alternative is most appropriate for the situation. Its primary objective is to improve teenagers’ emotional intelligence skills such as self-esteem, assertiveness and decision making, in order to manage their social life improving health and well-being and avoiding addictions and violence. Program sessions are detailed in Table 2.

### 2.4. Statistical Analysis

First, a multivariate analysis of variance (MANOVA) was performed with total pretest scores from the variables included in the study in order to confirm the possible pretest difference in the variables, as a whole, between experimental group participants and control group participants. Second, in order to determine the program’s effect, descriptive and variance analyses (ANOVAs) were carried out with each one of the scores obtained for the instruments used during the pretest phase. Third, having confirmed the homogeneity of the two groups a priori, and in order to determine whether the change was significantly different in experimental group versus control group participants, a multivariate analysis of covariance (MANCOVA) was performed on the study’s variables as a whole. Besides, descriptive analyses and analyses of covariance were performed on posttest scores (posttest ANCOVAs co-varying for pretest scores), in order to analyze the impact of the program for every variable of the study. Lastly, the effect size was analyzed (Cohen’s d) (small < 0.50; moderate 0.50–0.79; large ≥ 0.80) [67].

## 3. Results

Results obtained in the pretest or basal evaluations are first presented, followed by the results for evaluating the impact of the “Aislados” Program in the variables studied. 

The pretest MANOVA results did not reveal statistically significant differences between the groups prior to the intervention, Wilks’ Lambda, Λ = 0.472, F (5182) = 0.742, *p* = 0.241, with a small effect size (η^2^ = 0.088, r = 0.10).

Results from the ANOVAs in the pretest phase (see Table 3) showed that before initiating the “Aislados” Program there were no statistically significant differences between experimental and control groups in any of the variables studied.

Results from the pretest-posttest MANCOVA revealed significant differences between the two conditions, Wilks’ Lambda, Λ = 1.414, F (5, 182) = 4.373, *p* = 0.004, with an average effect size (η^2^ = 0.348, r = 0.30). 

Analyses of covariance (ANCOVAs) were subsequently performed on the pretest-posttest scores in each one of the variables in the experimental and control groups. The results are shown in Table 3. As it can be seen, the results from the posttest ANCOVAs (co-varying the pretest scores) showed that participants in the experimental group significantly increased scores in Health-Related Quality of Life, Positive Affect and Mental Health. In addition, a small effect size was found for Health-Related Quality of Life (*d* = 0.22), Positive Affect (*d* = 0.38) and Mental Health (*d* = 0.29).

## 4. Discussion

The present study analyses the effects of video game intervention program called “Aislados” for the improvement of SWB, mental health and trait emotional intelligence among a sample of Spanish adolescents of middle and high school age. There is a growing body of literature on SEL intervention programs; these studies provide evidence that SEL competencies can be beneficial to students, whether in or out of school [68]. However, there is limited evidence about the impact of intervention programs by using video game and develop this kind of competencies in learning environments among adolescents and young people.

Results showed statistically significant differences between adolescents that received the intervention compared to those that did not participate in the program. Differences between both groups refer to some variables that were assessed. Then, the findings show that “Aislados” produced improvements in experimental group participants in terms of the following variables: (1) a significant increase in health-related quality of life, related to the cognitive component of SWB (CWB); (2) a significant increase in positive affect, related to the affective component of well-being (AWB); (3) a significant increase in mental health; (4) There are no significant improvements in trait emotional intelligence.

First, these results support the effectiveness of the “Aislados” program in improving of some SWB components, partially confirming hypothesis 1. The results are consistent with those found in other studies which have shown efficacy of SEL programs to improve SWB in children and adolescents [1,3,5,15]. These findings provide empirical evidence that reinforce the fundamental purpose of the SEL in the promotion of the quality of life and well-being of people [8,12]. A possible explanation for these results may be that the training of socio-emotional skills is a necessary factor to improve SWB due to its benefits in psychosocial adjustment [15]. Furthermore, the results confirm an improvement in positive affectivity, while the program has not had a positive impact on the reduction of negative affectivity. The “Aislados” program is aimed at improving social and emotional skills understood as protective resources, but reducing negative factors is not a specific aim of this program. In fact, positive and negative affect are independent dimensions; then, the reduction of NA does not depend on the increase of PA [69,70]. In the same way, these positive results in SWB are in line with others found in similar studies using video games [34,41]. A possible reason of these results is the inclusion of activities which are varied, attractive and contextualized to the educational stage it is aimed for in the “Aislados” videogame. Last, we believe that group activities and exchange among participant students may have a very positive influence in the results. 

Second, the results confirm hypothesis 2 and reveal that the “Aislados” program would improve participants’ mental health (MH). These findings are similar to those found in various meta-analysis and research studies that show improvements in mental health, as well as the reduction of emotional problems through the implementation of SEL-based programs [1,8,9]. From our point of view it is likely that the subjective perception of mental health is a factor related to SWB and the improvement of certain emotional problems and, therefore, it is logical to think that both variables can be susceptible to improvement through this type of interventions.

Finally, the results fail to confirm hypothesis 3, which stated that the “Aislados” program would improve participants’ trait EI (TEI). The data do not match that obtained in other studies finding that EI increases when students participate in a video game [40,42]. These results can be explained by the characteristics of the program, because it does not specifically aim to the development of EI, although it includes tasks and sessions about this matter. However, the “Aislados” video game requires that the players can interpret the emotions of the different characters in the situations to which they are exposed. In this sense, Teique-ASF, is a short version that offers a global score and, therefore, does not allow assessing the different dimensions of the IE independently; thus, the understanding of emotions has not been able to be evaluated accurately.

It is important to point out some research limitations; first, regarding the generalizability of the results, obtained in a specific sample of secondary students from Spain. Furthermore, future studies should consider involving university students. Also, the study could be replicated in international contexts. In sum, although preliminary but promising, further research with the “Aislados” program is required to replicate and extend these findings and to test its application with larger and more diverse samples of students. 

A limitation of the present study refers to the critique of the heterogeneity of interventions that claim to be based on SEL and the possible comparison of results [71]. This means that research to promote the efficacy of SEL should be interpreted with caution due to the differences between them. However, our results confirm that through the “Aislados” video game based on SEL the quality of life, positive affect and mental health (crucial aspects in SEL) of the participating students have been improved.

In addition, it is advisable to carry out a long-term follow-up evaluation in order to examine whether the “Aislados” program effects were maintained over time [13,72].

Despite of limitations, the results show that the program produced a positive effect, promoting an increase in SWB and providing benefits in the quality of life related to health, positive affect and mental health in adolescents. Regarding the future lines of research, it would be convenient to evaluate the effect of “Aislados” on other variables such as socio-educational adaptation, social competence, interpersonal relationships or academic performance. For future studies, it would be necessary to examine the Aislados program’s effects on health-related quality of life (HRQOL) in line with other findings related to internet addiction and other online activities [73].

Likewise, it would be advisable to consider the possibility of investigating the influence of socio-emotional competencies and the psychological characteristics of the teachers who apply the intervention program.

## 5. Conclusions

In view of the results, we want to highlight the importance of implementing programs during adolescence and youth to promote emotional and behavioral adjustment to improve both personal growth and well well-being. Those results can also improve academic performance and prevent/reduce of risky personal behaviors and act as protective resources for coping daily demands. Those are conclusions related to the significant increase of SWB, mental health and trait emotional intelligence that has been proved by the analytical study.

Taken together, the findings reported in this study mainly support the importance of implementing programs to promote crucial social and personal competencies in the citizens of the future. Education administrators need to apply innovative strategies for teaching those new competencies related to social and emotional skills. In this regard, “Aislados” video game and its related intervention program have been proved as an effective educational strategy for that purpose. This educational program has been implemented in several educational activities in and out of school and is currently implemented in two countries Spain and Dominican Republic in the context of educational projects. Empirical evaluations of these experiences are being held to a better understanding on how this can help students belonging to different cultural contexts to develop skills related to SEL and to prevent health problems.

## Figures and Tables

**Table 1:** Reliability evidences.

Measures	α	CR	AVE	Ω
KIDSCREEN	0.88	0.87	0.681	0.89
SWLS	0.81	0.83	0.501	0.82
PANAS-PA	0.79	0.75	0.593	0.76
PANAS-NA	0.73	0.75	0.588	0.75
MH-5	0.72	0.72	0.514	0.71
TEIQue-ASF	0.89	0.88	0.694	0.91

*Note.* α = Cronbach’s Alpha; CR = Composite reliability, AVE = Average Variance Extracted; Ω = McDonald’s Omega index.

**Table 2:** Sessions Schedule.

Dimensions	Sessions
Dimension 1: Relationships	Sessions 1, 2, 8, 14 and 15: Introduction to the program Knowledge of interpersonal communication skills Importance of relationships Reflection on the forms of relationship with others
Dimension 2: Assertiveness	Sessions 3, 5, 13 and 16: The communication of emotional states to others Adaptive expression of emotions and thoughts Empathy skills Knowledge of the different assertive techniques
Dimension 3: Self-esteem	Session 2, 3 and 4: Importance of self-knowledge. Improve of self-image. Development of emotional well-being strategies.
Dimension 4: Decision making	Session 6, 7, 10 and 11: Emotional management strategies of the others Promotion of positive expectations in daily tasks
Dimension 5: Emotional Intelligence	Session 17, 18, 19 and 21: Emotional expression Emotional self-regulation Emotion regulation abilities and social Interaction Knowledge about the most effective emotional regulation strategies
Dimension 6: Addictions	Session 17, 18, 19 and 21: Drug definitions Consequences of teenage drug addiction Use/abuse of technologies
Dimension 7: Conflict management	Session 25, 26, 27 and 28: Assertive communication Divergent and convergent thinking skills Conflict as opportunity for change Conflict resolution

**Table 3:** Pretest and posttest measures for experimental and control groups.

	Pretest					Postest				
Variables	Experimental	Control				Experimental	Control			
	Media (DT)	Media (DT)	*F*	*p*	*d*	Media (DT)	Media (DT)	*F*	*p*	*d*
KIDSCREEN										
HRQL	34.99(5.81)	35.01(5.92)	1.327	0.599	0.01	36.69(6.03)	35.44(5.38)	1.999	0.038	0.22
SWLS										
SWL	3.97(0.91)	4.03(0.87)	0.641	0.732	0.07	3.98(0.82)	3.99(0.92)	0.584	0.638	0.01
PANAS										
PA	21.23(3.73)	21.02(4.08)	1.543	0.196	0.05	22.25(3.01)	21.10(2.98)	2.932	0.041	0.38
NA	12.03(3.92)	11.97(3.73)	0.871	0.723	0.02	11.99(3.83)	11.84(3.97)	1.372	0.127	0.04
MH-5										
MH	15.98(5.12)	15.72(5.26)	0.756	0.723	0.05	17.14(4.21)	15.82(4.94)	2.146	0.025	0.29
TEIQUE-ASF										
TEI	4.85(0.62)	4.91(0.55)	1.986	0.217	0.10	4.87(0.59)	4.85(0.53)	1.413	0.433	0.04

Note. (1) HRQL: Health-Related Quality of Life; SWL: Satisfaction with Life; PA: Positive Affect; NA: Negative Affect; MH: Mental Health; TEI: Trait Emotional Intelligence; (2) *p*= *p*-value; (3) *d* = Cohen´s effect size. (4) Experimental group: *n* = 97, Control group: *n* = 90.
